# Oregano Phytocomplex Induces Programmed Cell Death in Melanoma Lines via Mitochondria and DNA Damage

**DOI:** 10.3390/foods9101486

**Published:** 2020-10-17

**Authors:** Valentina Nanni, Gabriele Di Marco, Gianni Sacchetti, Antonella Canini, Angelo Gismondi

**Affiliations:** 1 Department of Biology, University of Rome “Tor Vergata”, Via della Ricerca Scientifica 1, 00133 Rome, Italy; nanniva@gmail.com (V.N.); gabriele.di.marco@uniroma2.it (G.D.M.); canini@uniroma2.it (A.C.); 2 Terra&Acqua Tech-Research Unit 7, Pharmaceutical Biology Lab, Department of Life Sciences and Biotechnology, University of Ferrara, Piazzale Luciano Chiappini 3, 44123 Ferrara, Italy; scg@unife.it

**Keywords:** oxidative stress, necroptosis, plant extract, secondary metabolite, γH2AX, copper

## Abstract

Plant secondary metabolites possess chemopreventive and antineoplastic properties, but the lack of information about their exact mechanism of action in mammalian cells hinders the translation of these compounds in suitable therapies. In light of this, firstly, *Origanum vulgare* L. hydroalcoholic extract was chemically characterized by spectrophotometric and chromatographic analyses; then, the molecular bases underlying its antitumor activity on B16-F10 and A375 melanoma cells were investigated. Oregano extract induced oxidative stress and inhibited melanogenesis and tumor cell proliferation, triggering programmed cell death pathways (both apoptosis and necroptosis) through mitochondria and DNA damage. By contrast, oregano extract was safe on healthy tissues, revealing no cytotoxicity and mutagenicity on C2C12 myoblasts, considered as non-tumor proliferating cell model system, and on *Salmonella* strains, by the Ames test. All these data provide scientific evidence about the potential application of this food plant as an anticancer agent in in vivo studies and clinical trials.

## 1. Introduction

More than one-third of all pharmaceutical molecules approved by the Food and Drug Administration and by the European Medicines Agency are natural compounds, or their derivatives, and about one-quarter of them specifically originate from plants [1,2,3,4]. In detail, over 60% of the anticancer drugs are phytochemicals, such as alkaloids and polyphenols, confirming that the plant kingdom is a valuable source of chemopreventive and chemotherapeutic agents [5,6,7].

Overall, scientific data have documented that this type of metabolites exerts an inhibitory effect on a broad range of mammalian tumor cell lines in in vitro and in vivo systems [8,9]. According to the literature, the main mammalian cellular and molecular mechanisms influenced by plant molecules are those that involve the following targets: nuclear factor kappa-light-chain-enhancer of activated B cells (NF-κB); protein tyrosine kinases (PTKs); target mitogen-activated protein kinases (MAPKs); cyclooxygenase (COX-2); cyclin-dependent kinases (Cdks); phosphoinositide 3-kinase (PI3K) interactors; activator protein 1 (AP1); and cytoskeleton components [10,11,12,13,14,15,16,17,18,19,20,21,22].

Among all biological properties, the free radical scavenging activity seems to be the most validated device employed by phytochemicals to inhibit tumor cell growth, counteracting reactive oxygen species (ROS) production and limiting protein, lipid, and DNA oxidation [23]. Nevertheless, a growing body of evidence brought this opinion into question, arguing that the antiradical activity of the natural compounds has a potential role in chemoprevention, but it cannot fully explain the relative antitumor effect [24,25]. In addition, it is important to underline that a wide number of plant secondary metabolites have also shown unexpected pro-oxidant consequences, including DNA damage and apoptosis, especially at high concentrations and in the presence of transition metal ions [26,27,28,29].

*Origanum vulgare* L., also known as oregano, is a Mediterranean plant species belonging to the Lamiaceae family which, nowadays, represents one of the most used culinary herbs. However, the application of oregano in several ethnobotanical practices, including folk medicine, dates to ancient times. Regarding its phytotherapic effect, various investigations have been performed, documenting that oregano essential oil possesses antimicrobial, antiviral, antifungal, antioxidant, anti-inflammatory, digestive, expectorant, neuroprotective, antispasmodic, and antidiabetic properties, simultaneously. Moreover, some literature works have also associated a strong anticancer activity to such type of plant extract. For these reasons, *O. vulgare* is capturing greatly the attention of the food, cosmetic, and pharmaceutical industries [30,31,32,33,34,35].

According to all this evidence, the present research aimed at investigating the molecular mechanism underlying the antineoplastic effect of *O. vulgare* L. ssp. *hirtum* phytocomplex against murine (B16–F10) and human (A375) melanoma cells.

## 2. Materials and Methods

### 2.1. Plant Material

*Origanum vulgare* L. plants were collected at the *Vatopedi Holy Monastery* on Mount Athos (Greece), in the summer of 2018. The plant material was transferred to the Botanical Garden of Rome “Tor Vergata”, where its taxonomic identity was confirmed by Prof. Antonella Canini and Prof. Angelo Gismondi, based on morphological features. A part of the sample was deposited in the *Herbarium* of the Botanical Garden (voucher n. 127C), while the remaining portion was dried out (for 7 days at 37 °C) and used for the present research. In particular, the whole dried plants were powdered in liquid nitrogen, resuspended in 50% ethyl alcohol (200 mg/mL) and incubated, in agitation, for 24 h in the dark. After centrifugation for 20 min at 11,000 g, the supernatant was filtered (0.22 μm), completely desiccated at 30 °C by a vacuum drying system (Concentrator Plus, Eppendorf, Hamburg, Germany), and stored at −80 °C.

### 2.2. Total Phenol and Flavonoid Content

Hydroalcoholic oregano extract (HCOE) was solubilized in 50% ethyl alcohol at the final concentration of 200 mg/mL. The phenolic content in HCOE was measured according to the Folin–Ciocalteu modified method, as described in Impei et al. [36]. Results were reported as µg of gallic acid equivalents per gram of dried plant material (μg GAE/g DMW), applying a gallic acid calibration curve (0–30 mg/L). The amount of flavonoids in OE was assessed by the aluminium chloride colorimetric method [37]. Data were reported as µg of quercetin equivalents per gram of dried plant material (μg QE/g DMW), using a quercetin calibration curve (0–50 mg/L).

### 2.3. High-Performance Liquid Chromatography-Diode Array Detector (HPLC-DAD) and Gas Chromatography-Mass Spectrometry (GC-MS) Analyses

HCOE was characterized by an HPLC system (Shimadzu, Kyoto 604-8511, Japan) associated with an SPD-M20A diode array detector (DAD, Shimadzu, Kyoto 604-8511, Japan) and a Phenomenex Luna C18(2) (3 µm × 4.6 mm × 150 mm) column. A flow of 0.95 mL/min was applied, using formic acid 1% (buffer A) and methanol (buffer B). The following elution gradient was adopted: t_0 min_ (A 85%, B 15%); t_20 min_ (A 65%, B 35%); t_55 min_ (A 10%, B 90%); t_68 min_ (A 85%, B 15%); t_70 min_ (A 85%, B 15%). UV–visible absorption spectra at 280 and 340 nm were monitored. Plant metabolites were identified and quantified comparing their retention time, absorbance spectrum, and chromatographic peak area with those of relative pure standards (Sigma-Aldrich) at different concentrations. The amount of each detected molecule was reported as µg per 100 mg of dried plant material (µg/100 mg DMW).

GC-MS analysis was performed exactly as described in Nanni et al. [29]. In particular, to perform this investigation, HCOE was solubilized in 100% methanol at the final concentration of 200 mg/mL and then injected in the instrument.

### 2.4. Cell Cultures and Plant Treatments

Murine melanoma cells (B16–F10), human melanoma cells (A375), and murine myoblasts (C2C12) were cultured under standard conditions [38,39] in Dulbecco’s Modified Eagle’s Medium (DMEM). For cell experiments, HCOE was solubilized in sterilized PBS 1X, at the concentration of 1200 mg of dried plant material equivalent per mL, and added directly to the cell medium at specific doses. In particular, treatments were performed by exposing cells, for 4, 24 or 48 h, at 2, 4, 6, 8, or 10 mgs of dried plant material equivalent per mL of culture medium. Control cells (CNT) were treated only with PBS, at the highest volume of treatment, to check the influence of this non-toxic solvent on cells.

### 2.5. Cell Proliferation, Selectivity Index, and Cell Cycle Analysis

Cell viability was evaluated by the MTT kit (Sigma-Aldrich Merck, Darmstadt, Germany), as reported in the relative guidelines. Results were reported as percentage variation compared to the control (CNT), which was considered as a unit (100%). Plant extract cytotoxicity was measured using the Trypan Blue (1%, *w*/*v*) exclusion test and counting dead cells by a Neubauer-modified chamber. The selectivity index (SI) of HCOE on tumor and non-tumor cells was measured according to the following formula: SI = IC_50_ non-tumor cell line/IC_50_ cancer cell line (considering that IC_50_ represented the concentration at which 50% of cell proliferation was inhibited). Cell cycle analysis was performed by a FACSCalibur instrument (Beckton and Dickinson, Le Pont-de-Claix, France) associated to CellQuest software, counting 10,000 events per sample and using the protocol documented in Gismondi et al. [40]. Cytofluorimetric data were shown as a percentage of cells in G0/G1, S, G2/M, and sub-G1 phase. Other treatments were performed by necrostatin-1 (NEC-1; 20 μM, 48 h), Z-VAD-FMK (Z-VAD; 20 μM, 48 h), and Paclitaxel (TAX; 20 nM, 8 h) (Sigma-Aldrich).

### 2.6. Mutagen and Mutagen-Protective Activity

An Ames test was carried out on *Salmonella typhimurium* strains (TA97a, TA98, TA100 and TA1535) in order to evaluate the mutagen and mutagen-protective activity of HCOE. The assays were carried out as widely reported by Rossi et al. [41,42]. In detail, the mutagenic activity was determined by counting *Salmonella* colonies (Colony Counter 560; Suntex Instruments Company Ltd., New Taipei City, Taiwan) in plates treated with different concentrations of oregano extract in the presence and absence of S9 mix metabolic activation. The results were considered positive (potential mutagen) if the amount of revertant colonies was, at least, double that of the negative control. To determine the potential mutagen protection capacity of HCOE (concentration range: 0.01–0.1 mg/plate), bacteria were exposed to mutagenic agents, with or without S9 mix, and exposed or not to different concentrations of HCOE. Used mutagens were 2-nitrofluorene (2 μg/plate; Sigma-Aldrich) for TA97a, TA98, and TA1535; NaN_3_ (2 μg/plate; Sigma-Aldrich) for TA100 without S9; and 2-aminoanthracene (2 μg/plate; Sigma-Aldrich) for all *Salmonella* strains cultured with S9 mix. Data were expressed as CFU/plate. The inhibition rate (IR) of HCOE for mutagenic induction was measured according to the formula: IR (%) = (A−B) × 100/A (where A and B represent the number of revertants in positive controls or in plates with HCOE, without spontaneous colonies, respectively). Negative controls, represented by dimethylsulfoxide (DMSO) treated strains, were performed to evaluate the background of spontaneous revertants.

### 2.7. Protein Analysis

Cells were lysed in High Salt Buffer (2 mM CaCl_2_, 350 mM KCl, 50 mM Tris HCl pH 7.4, 1 mM MgCl_2_) containing 1% protease inhibitor cocktail and 1% NP40. Proteins whose concentration was measured by the Bradford method [43] were separated by SDS-PAGE and transferred onto nitrocellulose membrane. Protein signals were detected by a chemiluminescent kit (Luminol Reagent; Santa Cruz Biotechnology, Dallas, TX, USA) and a VersaDoc Imaging System associated with Quantity One software (Bio-Rad). After normalization with GAPDH, the results were indicated as percentage change compared to the CNT, which was considered as a unit (100%). The antibodies (Santa Cruz Biotechnology) used for Western blotting analyses were as follows: mouse monoclonal GAPDH; mouse monoclonal microphthalmia-associated transcription factor (Mitf); mouse monoclonal p53; mouse monoclonal Parp-1; mouse monoclonal caspase-3 (Casp-3); rabbit polyclonal Bcl-2; rabbit polyclonal Bax; mouse monoclonal control outer mitochondrial membrane protein TOMM20; mouse monoclonal cytochrome c (Cycs); peroxidase-conjugated rabbit, and mouse secondary antibodies. Staurosporine (STS; 2 μM, 4 h) was used as an inducer for apoptosis (Sigma-Aldrich).

### 2.8. Real-Time-PCR (RT-PCR) Assay

Total RNA was extracted by a Pure Link RNA Mini Kit (Invitrogen, Thermo Fisher Scientific, Waltham, MA, USA). RNA concentration and purity were evaluated with a Nanodrop ND1000 spectrophotometer (Thermo Fisher Scientific, Waltham, MA, USA). For cDNA synthesis, 2.5 µg of RNA were incubated for 2 min at 65 °C with 0.4 mM of each dNTP (Euroclone, Milan, Italy). Then, 40 units of RNA inhibitor (Promega, Madison, WI, USA), 0.5 µg random hexamer primers (Invitrogen, Thermo Fisher Scientific, Waltham, MA, USA), 200 units of Moloney murine leukemia virus reverse transcriptase (Promega), 1× enzyme buffer, and 10 mM dithiothreitol were added to reach the final volume of 25 µL. The mix was incubated for 90 min at 37 °C. RT-PCR reactions were carried out in 20 µL of volume composed of 10 ng of cDNA, 5 µM of each primer, and 50% SYBR green (Kapa SYBR Fast qPCR kit; Kapa Biosystems, Roche, Wilmington, MA, USA, Country). cDNA amplification was carried out in an IQ5 thermocycler (Bio-Rad) with the following method: (i) initial denaturation at 95 °C, 4 min; (ii) 45 cycles of denaturation at 95 °C for 20 s (sec), primer annealing at 60 °C (for all genes) for 30 s, and extension at 72 °C for 30 s; and (iii) production of disassociation curve, from 50 to 90 °C (rate: 0.5 °C every 5 s), for the verification of the results. The 2^-ΔΔCt^ formula was used to measure mRNA concentration for each gene: in detail, the threshold cycle (Ct) of the target gene monitored in the treated sample was normalized for the internal reference gene (β-actin, ACTB; ΔCt) and for the respective value of the control sample (ΔΔCt), which was considered as a unit (100%). Supplementary Materials Table S1 reports the list of primers used in this work: microphthalmia-associated transcription factor (MITF), tyrosinase-related protein 1 (TYRP1), tyrosinase (TYR), P21, P27, P53, cyclin-dependent kinase 1 (CDK1), cyclin B1 (CCNB1), and β-actin (ACTB) [44,45,46,47,48].

### 2.9. Reactive Species Level and Mitochondrial Mass and Membrane Potential Measurement

Intracellular reactive oxygen (ROS) and nitrogen (RNS) species, mitochondrial mass, and mitochondrial transmembrane potential were measured by 2′,7′-dichlorodihydrofluorescein diacetate (DCFH-DA; green signal; 10 μM, 15 min), 4-amino-5-methylamino-2′,7′-difluorofluorescein diacetate (DAF-FM DA; green signal; 2.5 μM, 30 min), MitoTracker Green (MTG; green signal; 250 nM, 30 min), and MitoTracker Red CMX ROS (MTR; red signal; 250 nM, 30 min) fluorescent assays (Sigma-Aldrich), respectively. The analyses were performed using the protocol described in Gismondi et al. [49] (FACSCalibur instrument; filters: FL-1^+^ for green; FL-2^+^ for red) and counting 10,000 cytofluorimentric events per sample. Negative controls were carried out treating cells with PBS 1X, whereas positive controls were produced incubating cells with hydrogen peroxide (H_2_O_2_; 5 mM; for DCFH-DA test), S-nitrosoglutathione (GSNO; 0.5 mM: for DAF-FM DA test), and carbonyl cyanide m-chlorophenyl hydrazone (CCCP; 10 μM; for mitochondrial tests) for 4 h before the exposure to the appropriate probe. All results were reported as a percentage variation of cell fluorescence compared to the CNT sample, which was considered as a unit (100%). Changes in the mitochondrial membrane potential were reported as MTR/MTG ratio, as suggested by Pendergrass et al. [50].

### 2.10. Immunofluorescence Microscopy

For γH2AX and 53BP1 foci detection, cells (grown on slides) were fixed in 4% paraformaldehyde for 15 min, permeabilized with 0.4% Triton X-100 in PBS for 10 min, blocked in PBS blocking solution (10% FBS, 0.1% Triton X-100) for 3 h, and incubated for 2 h with primary antibodies (mouse monoclonal γH2AX Ser-139; rabbit polyclonal 53BP1; Merck Millipore). Then, samples were exposed for 1 h to the respective secondary antibodies (goat anti-mouse IgG labeled with Alexa Fluor 488 and goat anti-rabbit IgG labeled with Alexa Fluor 594; Invitrogen, Eugene, OR, USA). Nuclei were stained with 0.1 mg/mL of DAPI for 1 min. Images were acquired by a Leica DMR microscope (Leica Microsystems, Wetzlar, Germany) equipped with a Leica DFC 350 FX digital camera, EBQ 100 isolated fluorescent lamp (Leistungselektronik Jena GmbH, Jena, Germany), UV/FITC/TRITC filters, and 40X and 100X objectives. All images were elaborated by Leica Qwin Pro image analysis software and captured at the same instrument settings and exposure times in order to ensure a correct comparison. For foci counting, 500 cells for each experimental condition were analyzed by ImageJ. Control treatments were performed by etoposide (ETO; 500 nM, 8 h) and triethylenetetramine (TETA; 50 µM, 48 h) (Sigma-Aldrich).

### 2.11. Statistical Analysis

Results were reported as mean value ± standard deviation (SD) of measurements obtained by independent experiments (*n* ≥ 3). Statistical significance was evaluated by one-way ANOVA test (Microsoft Excel software) vs. the respective control; a *p*-value < 0.05 was considered significant (* < 0.05; ** < 0.01; *** < 0.001).

## 3. Results

### 3.1. Chemical Characterization of the O. vulgare L. Phytocomplex

For a preliminary typization of the plant sample, the concentration of simple phenols and flavonoids in the *O. vulgare* L. extract was measured by spectrophotometric analyses. The amount of total phenols in HCOE was equal to 107.50 ± 10.81 μg GAE/g DMW, while flavonoids were 230.79 ± 13.97 μg QE/g DMW. Then, in order to identify the main plant metabolites underlying the bioactivity of the oregano extract, two different chromatographic approaches were applied to characterize the biochemical profile of this natural matrix. As reported in Table 1, 13 compounds were identified and quantified in HCOE by the HPLC-DAD technique (Supplementary Materials Figure S1).

The most abundant molecule was chrysin (8.47 ± 0.06 µg/100 mg DMW), followed by quercetin-3-*o*-arabinoside (2.37 ± 0.04 µg/100 mg DMW) and rutin (2.15 ± 0.05 µg/100 mg DMW). Moreover, the phytocomplex extracted by *O. vulgare* L. samples was characterized by GC-MS analysis; in total, 45 secondary metabolites were detected and subjected to relative quantitation (Table 2).

The most abundant molecules were carvacrol (34.82%), thymol (16.61%), and linolenic acid methyl ester (7.96%).

### 3.2. O. vulgare L. Extract Reduces B16-F10 Cell Growth Not Affecting C2C12 Cell Viability

**Figure 1:**
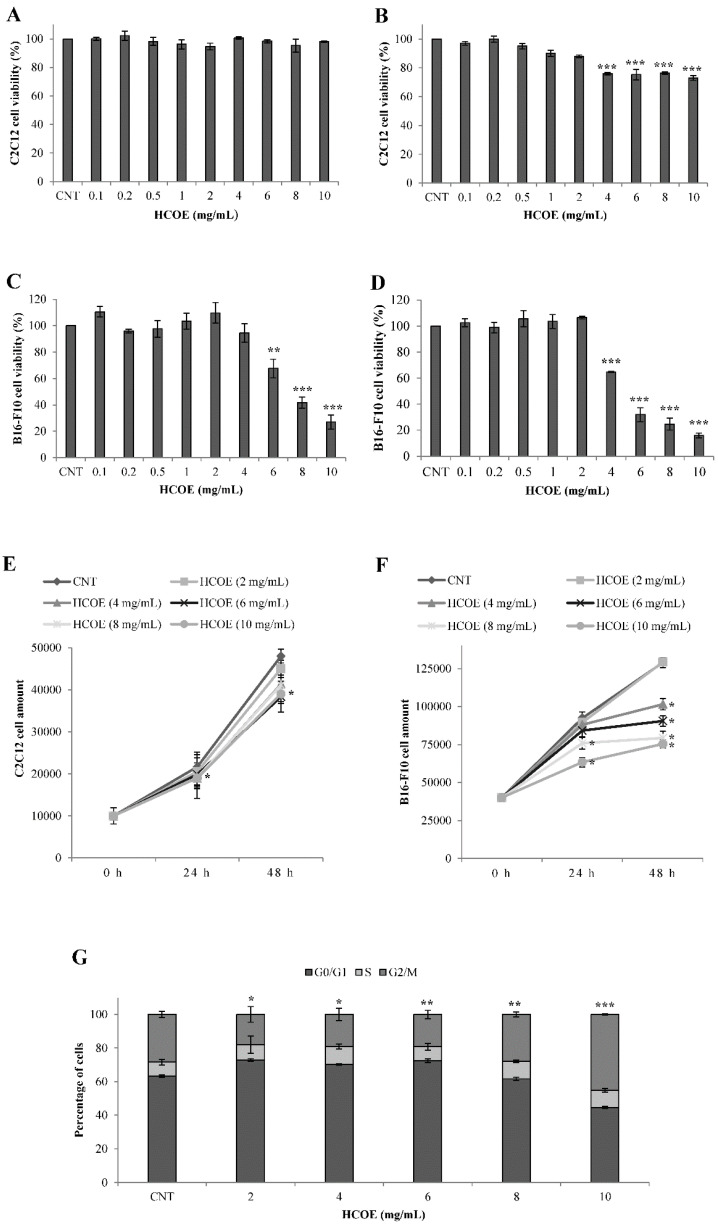
C2C12 and B16-F10 cell growth. C2C12 (**A**,**B**) and B16-F10 (**C**,**D**) cell growth was measured after 24 h (**A**,**C**) and 48 h (**B**,**D**) of treatments with Phosphate Buffered Saline (PBS), as control, and different concentrations of *O. vulgare* plant extract (HCOE). Results expressed as percentage with respect to PBS represent the mean ± SD of four independent experiments (* *p* < 0.01; *** *p* < 0.001 vs. control). Proliferation curves of C2C12 (**E**) and B16-F10 (**F**) cells were generated counting, by a Neubauer modified chamber, the amount of alive cells after staining with Trypan Blue at 0, 24, and 48 h of treatments with HCOE. Results are indicated as the mean ± SD of four independent experiments. (* *p* < 0.05 vs. control) (**G**) Cell cycle analysis of B16-F10 after treatment, for 48 h, with 2, 4, 6, 8, and 10 mg/mL HCOE is shown. For each sample, the percentage amount of cells in every cycle phase (G0/G1, S, and G2/M) was measured by cytofluorimetric analysis. Results are indicated as mean ± SD of four independent experiments (* *p* < 0.05; ** *p* < 0.01; *** *p* < 0.001 vs. control).

The biological effect of *O. vulgare* L. hydroalcoholic extract on the proliferation of B16-F10 cells, a murine melanoma line characterized by high aggressiveness and drug resistance, was investigated, by MTT assay, after exposure for 24 and 48 h with different concentrations of plant phytocomplex (0.1–10 mg/mL). Simultaneously, to check the safety of HCOE on non-tumor cells, C2C12 myoblasts were exposed to similar treatments. HCOE did not affect C2C12 cell growth after 24 h of incubation (Figure 1A), whereas a slight decrease of myoblast viability was observed after 48 h of treatment, reaching 27% at the highest concentration of extract (Figure 1B). By contrast, the oregano sample significantly decreased B16–F10 proliferation: in particular, after 24 and 48 h of incubation, 10 mg/mL of HCOE caused a reduction of melanoma cell viability of 73.42% (Figure 1C) and 84.11% (Figure 1D), respectively. According to these results, IC_50_ values for C2C12 cells treated with HCOE, for 24 and 48 h, were estimated to be 55.44 and 14.28 mg/mL, respectively. For B16-F10, these values were 7.23 and 4.72 mg/mL, in that order. Consequently, for HCOE, the selectivity index was 7.66 after 24 h of treatment and 3.03 at 48 h.

The cytotoxicity of the oregano extract was evaluated by the Trypan Blue exclusion test. C2C12 and B16-F10 proliferation curves were generated, counting living cells after treatment with HCOE (2–10 mg/mL) or PBS (CNT) for 24 and 48 h. Simultaneously, dead cells were also counted in each sample. As expected, C2C12 cell growth was slightly affected by HCOE (Figure 1E). The strongest cytotoxic effect (21.25%) was observed after 48 h of incubation with the highest dose of HCOE. However, in all cases, the percentage of dead cells was always lower than 22% (Table 3). For B16-F10, cell proliferation significantly decreased in a dose-dependent manner (Figure 1F), and a remarkable percentage of dead cells was achieved at 10 mg/mL of HCOE (42% and 44.75% after 24 and 48 h of incubation, respectively) (Table 3).

To confirm the previous data, the B16–F10 cell cycle was analyzed after exposure to HCOE for 48 h. As shown in Figure 1G, low doses of plant extract induced a cell accumulation in the G1/G0 phase, whereas 10 mg/mL of HCOE determined an accumulation of cells in the G2/M phase equal to 45.24%.

### 3.3. Oregano Treatment Shows Antiproliferative Activity Also on A375 Human Melanoma Cells

**Figure 2:**
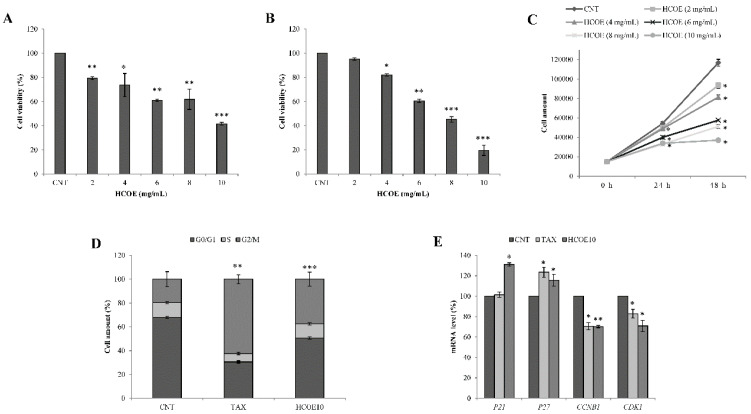
A375 cell growth and cycle. A375 cell viability was measured after 24 h (**A**) and 48 h (**B**) of treatment with 2, 4, 6, 8, and 10 mg/mL HCOE. Results, expressed as percentage with respect to the PBS control (CNT; considered as unit, 100%), represent the mean ± SD of four independent experiments (* *p* < 0.05; ** *p* < 0.01; *** *p* < 0.001 vs. control). (**C**) Proliferation curves of A375 cells were generated by counting, with a Neubauer modified chamber, the amount of alive cells after staining with Trypan Blue at 0, 24 and 48 h of treatment with HCOE. Results were indicated as mean ± SD of three independent experiments (* *p* < 0.05 vs. control). (**D**) A375 cell cycle analysis after 48 h of treatment with paclitaxel (TAX) or 10 mg/mL HCOE is shown. For each sample, the percentage amount of cells in every cycle phase (G0/G1, S and G2/M) was measured by cytofluorimetric analysis. Results are expressed as mean ± SD of three independent experiments (** *p* < 0.01; *** *p* < 0.001 vs. control). (**E**) P21, P27, CCNB1, and CDK1 mRNA levels, measured by RT-PCR, in A375 cells treated for 48 h with TAX or 10 mg/mL HCOE are reported. Gene expression, calculated as mRNA amount after normalization for ACTB mRNA (2^-∆∆Ct^), is reported as percentage with respect to the PBS control (CNT; considered as unit, 100%). Data represent the mean ± SD of three independent measurements (* *p* < 0.05; ** *p* < 0.01 vs. control).

The antiproliferative properties of the oregano extract on B16–F10 murine melanoma cells encouraged us to continue our research, testing if the same plant preparation could also exhibit similar effects on the A375 human melanoma line. The data obtained by the MTT assay are reported in Figure 2A,B. In detail, HCOE treatments at selected doses (2, 4, 6, 8, 10 mg/mL) decreased A375 cell viability, respectively, by 20.73%, 26.42%, 39.02%, 38.21%, 58.40%, and 3.86% after 24 h of incubation, and by 4.84%, 17.95%, 39.43%, 54.77%, and 80.40% after 48 h compared to the corresponding controls. In addition, in this case, the selectivity index was estimated. IC_50_ values for A375 cells treated with HCOE, for 24 and 48 h, were 9.14 and 7.08 mg/mL, respectively. Consequently, the SI with respect to C2C12 cells was 6.07 after 24 h of treatment and 2.02 at 48 h.

The Trypan Blue exclusion test confirmed these results, evidencing reduced proliferation curves (Figure 2C) and significant cytotoxicity levels on human melanoma cells, especially after exposure to 10 mg/mL HCOE (47.14% and 55.13% of dead cells after 24 and 48 h of incubation, respectively) (Table 3).

Taking into account the great antiproliferative activity exerted by HCOE, the A375 cell cycle was analyzed after treatment with 10 mg/mL of oregano extract for 48 h. As reported in Figure 2D, a significant increase of cells in the G2/M phase (19.55%) was detected with respect to the control (CNT). In this context, the (TAX), a well-known plant drug able to induce G2/M phase arrest [51,52], was also used as positive control.

This evidence was consistent with the RT-PCR experiments performed to measure cyclin-dependent kinase 1 (CDK1), cyclin B1 (CCNB1), and P21 and P27 mRNA levels (Figure 2E). Indeed, the expression of CCNB1 and CDK1 genes, which are key factors in the transition from the G2 to the M phase [53], appeared reduced after 48 h of incubation with oregano extract. At the same time, P21 and P27 transcripts, which are CDK1/Cyclin B1 inhibitors [54,55,56], increased in the presence of HCOE. Similar results were obtained after exposure to TAX, although the P21 mRNA level remained unaltered compared to the control, as documented by the literature [57].

### 3.4. Oregano Extract Has Neither Mutagenic Nor Mutagen-Protective Effects

Before investigating, in depth, the antitumor effect exerted by HCOE on A375 cells, the potential mutagenic and mutagen-protective activities of the oregano extract were analyzed in order to confirm HCOE safety on non-tumor living model systems. For the assessment of both properties, an Ames test was carried out with *Salmonella typhimurium* strains TA97a, TA98, TA100, and TA1535 in the presence or absence of the metabolic activator S9 mix. As indicated in Supplementary Materials Table S2, HCOE did not show any toxicological evidence. Indeed, at all tested doses, the t/c values, namely the ratio between the number of colonies of *Salmonella* strains grown in the presence of oregano extract (t) and those on the control medium (c), were never higher or equal to 2 and never presented a dose–response trend [58].

As concerns the evaluation of the mutagen-protection effect mediated by HCOE, a properly modified Ames test [40] was carried out. The plant extract did not exhibit any protective activity against well-known mutagen compounds, as indicated by the inhibition rate (IR) percentages reported in Supplementary Materials Table S3. Indeed, despite the presence of IR-positive values, the HCOE dose–response effect was not observed with significant values.

### 3.5. Oregano Phytocomplex Impairs MITF Pathway and Accumulates Reactive Species in A375 Cells

The molecular mechanism underlying oregano antineoplastic activity was clarified by using A375 cells as a model system. In particular, according to previous data, 10 mg/mL of HCOE (thenceforth HCOE10) was selected as a treatment dose, showing strong antiproliferative results on tumor cells and minimal effects on non-tumor ones.

**Figure 3:**
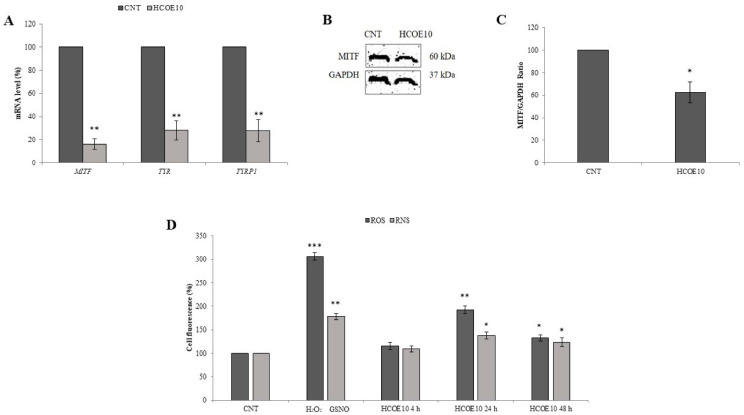
Analysis of mouse monoclonal microphthalmia-associated transcription factor (MITF) signaling and cell redox state. (**A**) *MITF*, tyrosinase (*TYR*)*,* and tyrosinase-related protein 1 (*TYRP1*) mRNA levels, measured by RT-PCR, in A375 cells treated for 48 h with 10 mg/mL HCOE are reported. Gene expression calculated as mRNA amount after normalization for β-actin (ACTB) mRNA (2^-∆∆Ct^) is reported as percentage with respect to the PBS control (considered as unit, 100%). Data represent the mean ± SD of four independent measurements (** *p* < 0.01 vs. control). (**B**) Representative Western blotting membrane of Mitf and Gapdh protein levels is shown. (**C**) Quantitation of MITF protein in A375 cells treated for 48 h with HCOE 10 mg/mL is reported. The results obtained by the ratio between Mitf and Gapdh (used as internal loading control) are indicated as percentage values with respect to PBS control (considered as unit, 100%). Data indicate the mean ± SD of three independent experiments (* *p* < 0.05 vs. control). (**D**) Intracellular reactive oxygen (ROS) and nitrogen (RNS) species levels were quantified in A375 cells, treated with 10 mg/mL of HCOE for 4, 24, and 48 h, by DCFH-DA and DAF-FM DA fluorescent assays, respectively. Radical species concentration is reported as percentage compared to PBS control (CNT). Hydrogen peroxide (H_2_O_2_) and S-Nitrosoglutathione (GSNO) treatments were performed as positive controls for ROS and RNS analysis, respectively. Results are expressed as mean ± SD of three independent measurements (* *p* < 0.05; ** *p* < 0.01; *** *p* < 0.001 vs. control).

The expression of the main genes involved in the MITF pathway, which is a cell signal that plays a crucial role in melanoma progression [59], was analyzed by RT-PCR (Figure 3A). In A375 cells, HCOE10 treatment, for 48 h, drastically decreased MITF, TYR, and TYRP1 mRNA levels of 16.27%, 27.93%, and 27.74%, respectively, compared to the control. This result was also corroborated by Western blotting analysis of Mitf protein content (Figure 3B); densitometric quantitation evidenced that oregano extract determined a reduction of 38.3% of Mitf protein, with respect to the control (Figure 3C).

**Figure 4:**
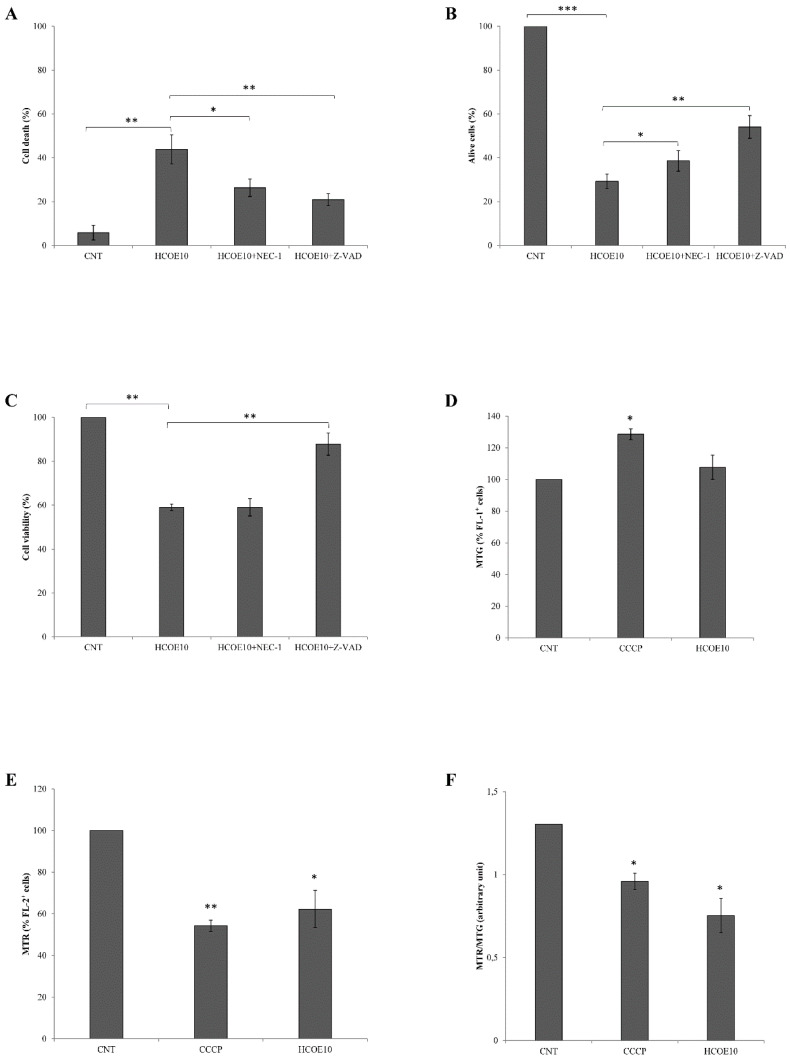
Cell death analysis and mitochondrial damage evaluation. A375 cell proliferation was analyzed after treatment, for 48 h, with 10 mg/mL HCOE, 10 mg/mL HCOE + 20 µM necrostatin-1 (HCOE10 + NEC-1), or 10 mg/mL HCOE + 20 µM Z-VAD-FMK (HCOE10+Z-VAD). (**A**) Cell death percentage was evaluated by flow cytometric assay, counting sub-G1 events. Results are expressed as mean ± SD of three independent experiments (* *p* < 0.05; ** *p* < 0.01 vs. control). (**B**) Alive cells were counted by the Trypan Blue exclusion test at 48 h of treatment with HCOE10, HCOE10+NEC-1, and HCOE10+Z-VAD. Results expressed as percentage with respect to the PBS control (CNT; considered as unit, 100%) are expressed as the mean ± SD of three independent experiments (* *p* < 0.05; ** *p* < 0.01; *** *p* < 0.001 vs. control). (**C**) A375 cell viability was measured by MTT assay after 48 h of treatment with HCOE10, HCOE10+NEC-1, and HCOE10+Z-VAD. Results, expressed as percentage with respect to the PBS control (CNT; considered as unit, 100%), represent the mean ± SD of three independent experiments (** *p* < 0.01 vs. control). Mitochondrial mass (**D**) and membrane potential (**E**) measurements, after 4 h of treatment with carbonyl cyanide m-chlorophenyl hydrazone (CCCP) (10 µM) (used as positive control) or 48 h of incubation with 10 mg/mL HCOE, were carried out by using MitoTracker Green (MTG) and MitoTracker Red CMX ROS (MTR), respectively. Results are expressed as percentage variation of fluorescence with respect to PBS control (CNT; considered as unit, 100%) (* *p* < 0.05; ** *p* < 0.01 vs. control). (**F**) Changes in mitochondrial membrane potential are expressed as MTG/MTR ratio (* *p* < 0.05 vs. control).

As the inhibition of the MITF pathway has been associated to reactive species burst [29], the influence of the plant extract (that is HCOE10 treatment for 4, 24 and 48 h) on ROS and RNS levels was monitored in A375 by DCFH-DA and DAF-FM DA assays, respectively (Figure 4D). Oregano treatment for 4 h did not influence cell redox state, whereas a prolonged exposure caused a significant increase of reactive species: after 24 and 48 h, respectively, +92.82% and +32.51% for ROS and +37.42% and +22.98% for RNS, compared to control cells. Positive controls, using inducers of ROS (i.e., H_2_O_2_) and RNS (i.e., GSNO), were carried.

### 3.6. Apoptosis/Necroptosis via Mitochondrial Pathway Is Induced in A375 Cells by HCOE

The decrease of melanoma cell growth, together with the evidence of high toxicity, cell cycle arrest, and MITF pathway inhibition, suggested that HCOE could induce cell death. For this reason, A375 cell viability was evaluated after co-treatments with oregano extract and Z-VAD-FMK (an anti-apoptotic pan-caspase inhibitor; [60]) or necrostatin-1 (NEC-1) (an inhibitor of necrosis/necroptosis [61]). Flow cytometry analysis (Figure 4A) showed that both co-treatments partially suppressed cell death (−17.43% of cells in the sub-G1 phase for the HCOE10 + NEC-1 sample; −22.90% for the HCOE10 + Z-VAD sample), with respect to the HCOE10-treated sample. Trypan Blue exclusion test (Figure 4B) confirmed this result, evidencing an increase of alive cells of 9.31% and 24.82% after exposure to HCOE10 + NEC-1 and HCOE10 + Z-VAD, respectively, compared to the treatment with only HCOE10. On the contrary, by MTT assay (Figure 4C), only HCOE10 + Z-VAD treatment seemed to rescue A375 cell viability compared to the HCOE10 sample.

Taking into account that mitochondrial damage is one of the main consequences of the oxidative stress (previously documented by ROS and RNS monitoring) and that the MTT assay (reported above) is an indicator of mitochondria activity [62,63], mitochondrial mass and membrane potential were estimated, respectively, by MitoTracker Green (MTG) and MitoTracker Red CMX ROS (MTR) cytofluorimetric assays in A375 exposed to HCOE10 for 48 h. CCCP, a well-known mitochondrial uncoupler, was used as a positive control. As shown in Figure 4D–F, HCOE10 treatment slightly affected mitochondrial mass, while it induced a strong depolarization. MitoTracker Red fluorescence was also normalized with the MitoTracker Green signal (MTR/MTG); this ratio was reduced by 42.17% after oregano treatment compared to the control.

**Figure 5:**
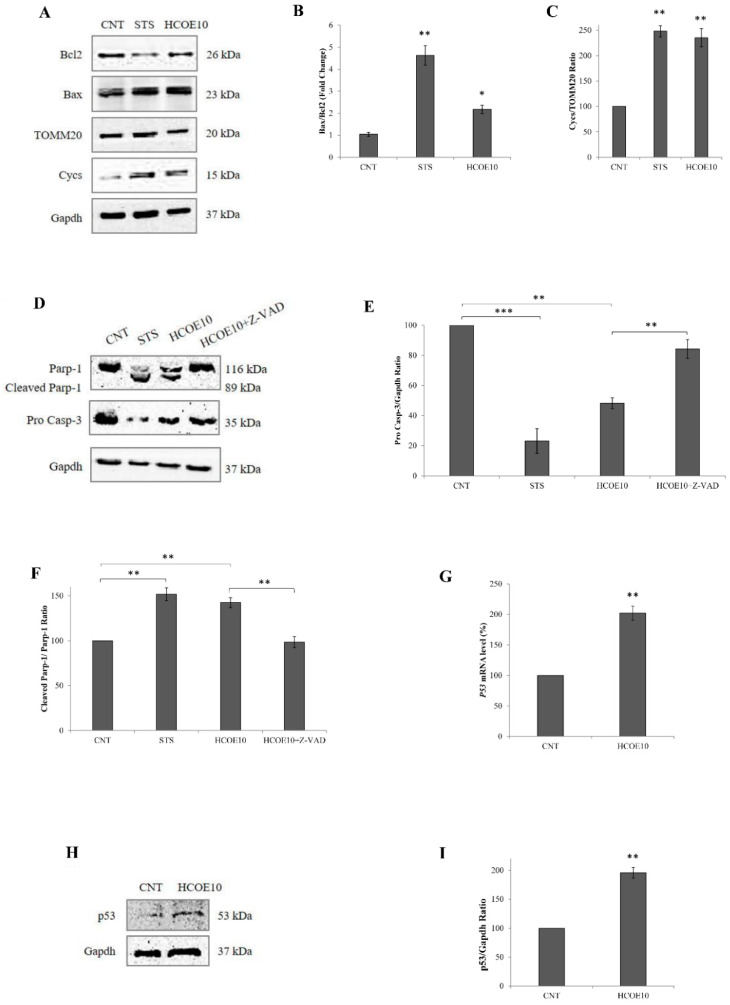
Analysis of mitochondria, apoptosis, and DNA damage markers. (**A**) Representative Western blotting membranes of Bcl-2, Bax, TOMM20, Cycs, and Gapdh protein levels evaluated in A375 cells treated for 4 h with staurosporine (STS, 2 µM) (used as positive control) or for 48 h with 10 mg/mL HCOE are shown. Quantitation of Bcl-2, Bax, TOMM20, and Cycs proteins was performed and the ratio between Bax/Bcl-2 (**B**) and Cycs/TOMM20 (**C**) are reported as percentage values, with respect to PBS control (CNT; considered as unit, 100%). (**D**) Representative Western blotting membranes of full length and cleaved form of Parp-1, pro caspase-3 (pro Casp-3), and Gapdh protein levels are shown. Quantitation of pro Casp-3 and Parp-1 proteins in A375 cells treated for 4 h with STS or for 48 h with 10 mg/mL HCOE and 10 mg/mL HCOE + 20 µM Z-VAD-FMK (HCOE10 + Z-VAD) are reported. (**E**) Pro Casp-3 levels evaluated by the ratio between pro Casp-3 and Gapdh (used as internal loading control) are expressed as percentage values with respect to PBS control (CNT; considered as unit, 100%). (**F**) The cleaved Parp-1/full-length Parp-1 ratio is expressed as percentage values with respect to PBS control (CNT; considered as unit, 100%). (**G**) P53 mRNA level were measured by RT-PCR in A375 cells treated for 48 h with 10 mg/mL of HCOE. Gene expression, calculated as transcript amount after normalization for ACTB mRNA (2^−∆∆Ct^), is reported as percentage with respect to the PBS control (CNT; considered as unit, 100%). (**H**) A representative Western blotting membrane of p53 and Gapdh protein levels is shown. (**I**) Quantitation of p53 protein in A375 cells treated for 48 h with 10 mg/mL HCOE is reported. Results obtained by the ratio between p53 and Gapdh (used as loading control) signals are indicated as percentage values with respect to PBS control (CNT; considered as unit, 100%). All data indicate the mean ± SD of three independent experiments (* *p* < 0.05; ** *p* < 0.01; *** *p* < 0.001 vs. negative control).

Mitochondrial membrane permeability loss suggested mitochondrial damage and apoptosis induction. To check this hypothesis, Western blotting analyses were carried out, monitoring specific markers of these phenomena. In particular, the protein levels of Bax, Bcl-2, cytochrome c (Cycs), and mitochondrial import receptor subunit TOMM20 were detected and quantified (Figure 5A–C). After 48 h of exposure to HCOE10, an approximately 2-fold increase of Bax/Bcl-2 ratio was observed. Moreover, as demonstrated in the case of staurosporin treatment (STS, used as positive control) [64], the plant extract caused the increase of Cycs (+135.2%) compared to TOMM20. The lack of changes in the TOMM20 level in oregano-treated cells confirmed the previous cytofluorimetric results for mitochondria mass.

Finally, additional immunoblots (and relative densitometric quantitations) were performed to study caspase-3 (Casp-3) and Parp-1 levels, as shown in Figure 5D–F. HCOE10 treatment, for 48 h, significantly decreased pro Casp-3 and full-length Parp-1 levels with respect to the negative control (CNT), inducing Parp-1 cleavage as also observed in the presence of STS. These effects were almost completely rescued by treating melanoma cells with HCOE10 and Z-VAD simultaneously.

### 3.7. HCOE Triggers DNA Breakages Mediated by Metal Ions

Since the pro-oxidant activity of several plant metabolites has been associated to DNA damage [65], the P53 gene expression level was monitored both in terms of transcript and protein amount, in A375 cells after exposure to HCOE10, for 48 h. As shown in Figure 5G–I, oregano treatment increased p53 mRNA (+102.1%) and protein (+95.95%) concentration, compared to the control.

**Figure 6:**
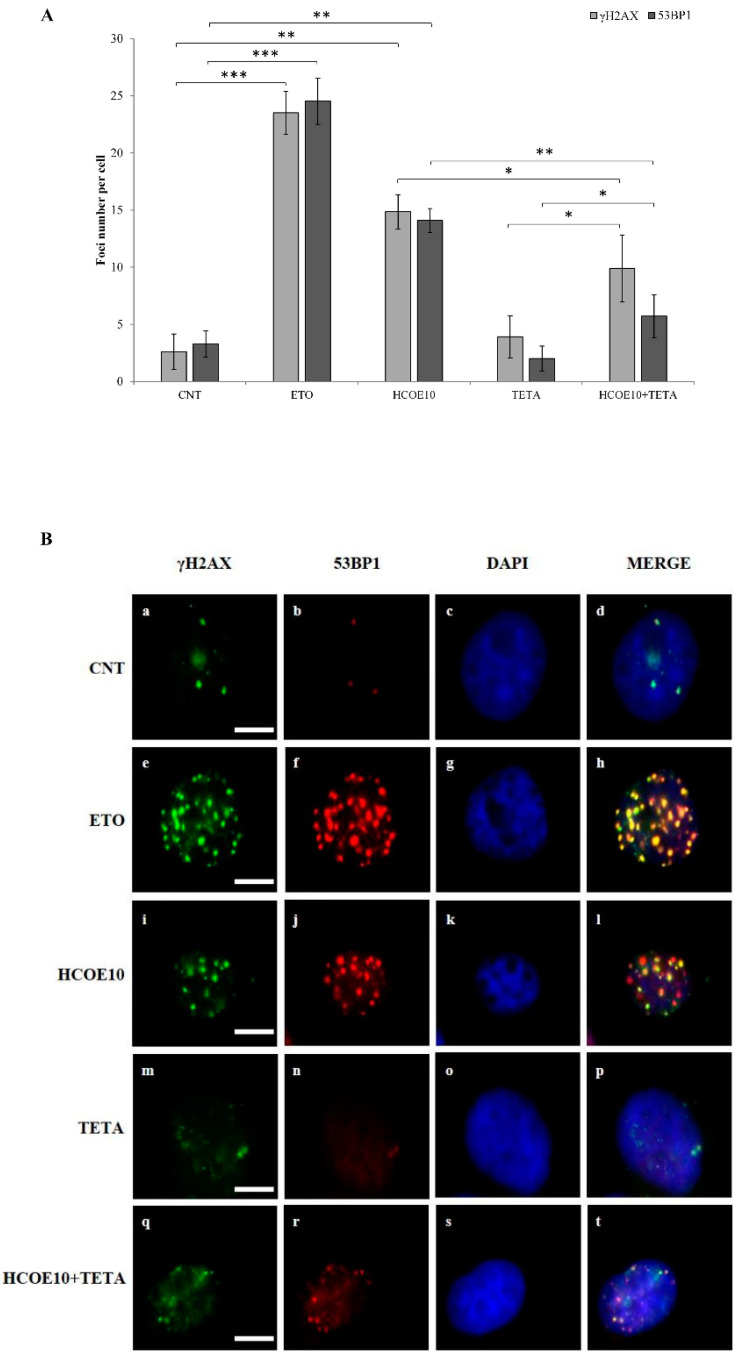
Immunofluorescence analysis. (**A**) The amount of γH2AX and 53BP1 foci detected in A375 cells treated with PBS (CNT), etoposide (ETO), 10 mg/mL HCOE (HCOE10), triethylenetetramine (TETA) or HCOE10+TETA were counted. Results are expressed as mean of foci per cell ± SD of three independent experiments (* *p* < 0.05; ** *p* < 0.01; *** *p* < 0.001 vs. control). (**B**) Representative immunofluorescence images of A375 cells treated with PBS (CNT) (**a–d**), etoposide (ETO) (**e–h**), 10 mg/mL HCOE (HCOE10) (**i**–**l**), triethylenetetramine (TETA) (**m**–**p**), or HCOE10+TETA (**q**–**t**) are shown. γH2AX foci are in green, 53BP1 foci are in red, while nuclei were stained in blue with DAPI. Merged images are also reported. The white bars indicate 15 μm.

To confirm the induction of DNA breakages by oregano extract, γH2AX and 53BP1 foci, two well-known markers of DNA damage [66,67], were detected by immunofluorescence (IF) analysis on A375 cells treated with HCOE10 for 48 h. In detail, an average of 14.86 ± 1.50 γH2AX foci and 14.10 ± 1.05 53BP1 foci per cell were measured with respect to 2.60 ± 1.56 γH2AX foci and 3.27 ± 1.14 53BP1 foci per cell found in the control sample (Figure 6A). Representative IF images per each sample were reported in Figure 6B; here, the treatment with etoposide (ETO), an inhibitor of topoisomerase II enzyme, represented the positive control. IF analysis showed that the major part (82% of cases) of γH2AX and 53BP1 foci co-localized (Figure 6B, panel l). In addition, considering that polyphenols, such as flavonoids, catalyze DNA breakages in the presence of metal ions (e.g., copper) [68], we evaluated the ability of oregano extract to trigger DNA damage in the presence of a copper chelator (TETA). The co-treatment HCOE10 + TETA determined a significant reduction of the level of DNA breakages (9.91 ± 2.91 γH2AX foci and 5.71 ± 1.87 53BP1 foci per cell) with respect to the pure treatment with HCOE10 (Figure 6A,B, panels q–t).

## 4. Discussion

Among tumors, skin cancer is the most common neoplasia worldwide. In particular, the more aggressive and deadliest form of this pathology is represented by melanoma [69]. Melanoma is a multi-factorial disease, depending on both environmental and endogenous factors. Indeed, about 90% of melanomas are caused by ultraviolet light exposure [70], while the remaining 10% has been associated to genetic defects [71]. Currently, such type of skin cancer is treated by surgical removal, which leads to a high survival rate except in the presence of metastases. In the latter case, a chemotherapeutic approach based on several drugs, such as Dacarbazine (an alkylating agent), Vemurafenib (BRAF kinase inhibitor), Ipilimumab (monoclonal antibody targeting for cytotoxic T-lymphocyte antigen-4), Pemrolizumab (monoclonal IgG4 antibody), and Nivolumab (monoclonal antibody targeting for Programmed Death-1 protein) [72], is the most efficient strategy to treat melanoma. However, the aggressiveness and the high rate of multi-drug resistance of this pathology highlight the need of new antineoplastic molecules.

Phytochemicals, namely secondary metabolites, produced by plants to protect themselves from environmental stresses and promote their reproduction [73], have been widely documented to exert a great variety of non-negligible bioactivities even on mammalian systems. Indeed, it has been documented that several plant compounds promote apoptosis and inhibit metastasis and angiogenesis [74,75,76,77]. For this reason, they have been taken under consideration thanks to their relevant medical and pharmaceutical properties. In this scenario, plant phytocomplexes can represent potential antiproliferative and anti-invasive cocktails for drug-resistant melanomas.

Based on the previous evidence, in the current research, the biological effect of a hydroalcoholic extract from plants of *Origanum vulgare* L. ssp. *hirtum* (HCOE) was investigated on highly metastatic and drug-resistant murine (B16-F10) and human (A375) melanoma cells. Indeed, although a potential antineoplastic effect has been associated to oregano extracts [30,31,32,33,34,35], the capacity of this herb to contrast the growth of the above-mentioned melanoma lines has never been elucidated in detail. Moreover, as one of the main goals of the cancer research is the discovery of new drugs with limited or without adverse side effects for healthy tissues, oregano extract was also tested on C2C12 myoblasts, which is a non-tumor cell model.

Since plant extract bioactivity cannot be attributed only to its more representative compounds, rather than to the synergic effect of plant molecules present both in high concentration and in trace [78,79,80], first of all, we investigated the biochemical profile of the oregano extract used in this study by chromatographic approaches (HPLC-DAD and GC-MS). A total of 58 metabolites were detected and recognized. Moreover, the amount of total phenols and flavonoids in HCOE was also measured, together with its in vitro antiradical power, to better characterize the plant extract.

The GC-MS chemoprofile obtained in the current research totally was in line with those documented in the literature. In this regard, *O. vulgare* ssp. *hirtum* essential oil can be classified in four chemotypes, according to its main constituents, especially thymol and carvacrol, and relative ratios. For instance, *O. vulgare* ssp. *hirtum* essential oil extracted from plants grown in Southern Italy and Northern Greece would seem rich in thymol, while that obtained from plant material propagated in Southern Greece was abundant in carvacrol [81,82,83]. Although purified from plants grown on Mount Athos (Northern Greece), our oregano extract showed a chemoprofile more similar to those of Southern Greece. To explain this phenomenon, it is important to keep in mind that several independent variables, such as plant growth stage and environment conditions, may strongly influence the phytocomplex. On the other hand, the oregano sample here studied revealed a content of phenolics double compared to that reported in the literature [84,85].

As concerns oregano biological activity, the plant phytocomplex determined a great reduction of B16-F10 cell growth, especially after 48 h of exposure with the highest doses (6, 8, 10 mg/mL), while it minimally influenced the myoblast division rate. B16-F10 and C2C12 proliferation curves confirmed the previous MTT outcomes. Moreover, HCOE induced a significant time- and dose-dependent toxicity on murine melanoma cells, whereas a low percentage of Trypan blue positive cells was detected in the case of C2C12. To validate these results, IC_50_ values and selectivity indexes (SI) for the plant treatments on both cell lines were calculated. According to the literature, a reliable SI value must be equal to or greater than 2 [86], and HCOE satisfied this requirement. Cell cycle analysis of B16-F10 demonstrated that oregano extract caused an increase of cells in G2/M phase.

This promising evidence encouraged us to check if oregano extract could exert antineoplastic effects also against a human melanoma cell line with the aim to lay the basis for future desirable application in translational medicine. As expected, significant antiproliferative activity, with a high selectivity index, together with a relevant cytotoxic effect of HCOE on A375 cells was confirmed.

Taking into account all previous data, the concentration of 10 mg/mL of oregano sample was selected for further experiments, showing the best antiproliferative effect.

Cytofluorimetric and RT-PCR analyses proved that the plant extract blocked cell division in the G2/M phase, acting on the expression of specific key genes implicated in the inception of the mitotic process (i.e., CDK1, CCNB1, P21, and P27) [53,55,56] such as paclitaxel, which is a well-known plant anticancer drug with antimitotic property.

Before proceeding with the other analyses, as the final objective of the current research was the valorization of an oregano hydroalcoholic extract for potential chemotherapeutic applications, the control of the safety for the plant preparation with reference to mutagenic properties was necessary. For this purpose, the Ames test, recommended by the European Food Safety Authority (EFSA) as a proper assay to assess food safety [87], was carried out. It provided the proof that HCOE did not have a non-mutagenic effect [88,89], as expected. Indeed, *O. vulgare* and its derivatives, which are accepted as food ingredients by the U.S. Food and Drug Administration, are listed among the GRAS (Generally Recognized As Safe) substances by the Code of Federal Regulations of the USA and are generally well tolerated by the human body, although gastrointestinal upset and skin allergic reactions have been associated to them [90]. However, the lack of data regarding oregano genotoxicity [91,92] highlights the need for further studies on this topic and valorizes the present preliminary results. The mutagen-protective activity of HCOE was also investigated in order to further analyze the plant extract under a healthy point of view. Unfortunately, at all tested doses, no protective property against known mutagenic compounds was documented.

In the second part of this work, the molecular mechanism underlying the bioactivity of HCOE on melanoma cells was investigated in depth. First of all, the efficiency of the MITF pathway was verified, studying the expression rate of MITF, TYR, and TYRP1 genes. The results revealed an antimelanogenic activity of the oregano extract on A375, as already suggested by the literature [93].

At low levels, reactive species play a key role in cell signaling, but their overproduction can lead to mitochondrial alterations (i.e., DNA mutations, respiratory chain damage, membrane permeability loss), oxidative stress, and an inhibition of specific genes, including those related to melanin synthesis [29,94]. Therefore, according to the previous results, intracellular ROS and RNS levels were measured in A375 cells, demonstrating that HCOE10 had a remarkable pro-oxidant effect, especially after 24 h of incubation. This evidence suggested that oregano antitumor activity could be based on a reactive species-mediated apoptotic process. Literature data about carvacrol bioactivity, one of the most abundant phenolic monoterpenoids of oregano (as also documented in the present research by GC-MS analysis, see Table 2), would support this hypothesis. Indeed, several published works showed that this compound induces apoptosis in different tumor cell lines by increasing the ROS amount and disrupting mitochondrial membrane potential [95,96,97].

All previous considerations suggested that HCOE10 could trigger cell death in A375. For this reason, to clarify which cell death pathway was induced by oregano extract, a pan-caspase inhibitor, Z-VAD-FMK, and an inhibitor of necroptosis, necrostatin-1 [98], were used in co-treatments with HCOE10 on the melanoma cells. The experiments demonstrated that the decrease of tumor cell proliferation was partially due to both apoptosis and necroptosis induction, as already suggested by Savini et al. [99] and Rubin et al. [100]. Indeed, these two phenomena are strongly correlated to each other because they share the same stimuli (such as TNF-α), ligands, and receptors [101,102]. The mechanism underlying the activation of apoptosis and/or necroptosis is still under investigation. Nevertheless, Annexin V/propidium iodide staining in the presence of necrostatin-1, as well as the analysis of TNF-α level, could be performed in the future to better clarify the role of these two pathways in oregano-induced cell death [103,104].

Since in MTT assay, only HCOE10 + Z-VAD double treatment rescued A375 viability, taking into account that mitochondria are both generators of and targets for reactive species (whose levels were previously observed to be increased in the presence of HCOE), a mitochondria impairment caused by oregano extract was hypothesized. To validate this theory, cytofluorimetric analyses, based on the use of MitoTracker Green and MitoTracker Red CMX-ROS probes, were performed. The data confirmed a loss of mitochondrial potential after HCOE10 treatment, while no significant change in mitochondrial mass was appreciated. By contrast, CCCP-treated cells (the positive control) showed an increase of mitochondrial mass, which was probably due to mitochondrial fragmentation [105,106] associated to an expected membrane depolarization.

As known in the literature, the permeabilization of the mitochondrial outer membrane is an event promoted by the pro-apoptotic protein Bax. Bcl-2, on the other hand, is an anti-apoptotic factor that prevents apoptosis by inhibiting Bax. Therefore, an elevated Bax/Bcl-2 ratio is a feature of apoptotis induction. As a consequence of Bax activation, damaged mitochondria release cytochrome c into the cytoplasm, leading to caspase-3 induction and Parp-1 cleavage [107,108]. In view of this, the amount of apoptosis-related proteins (Bax, Bcl-2, Cycs, Casp-3, and Parp-1) were evaluated in A375 after 48 h of incubation with HCOE10. Oregano extract was able to trigger apotosis in the human melanoma cells by increasing Bax and Cycs concentrations and decreasing Bcl-2 and pro Casp-3 amounts. Moreover, as expected, Parp-1 cleavage was evident in oregano-treated cells. HCOE10 + Z-VAD co-treatment reversed HCOE effect, showing protein levels similar to those detected in the respective negative control. All these results confirmed that the plant extract induced, in A375 cells, a caspase-dependent apoptosis, which was mediated by mitochondrial damage. However, considering that both ROS and RNS play an important role also in autophagy and that mitophagy is strictly involved in mitochondrial turnover [109,110], it would be interesting in the future to investigate if autophagy/mitophagy is also induced by oregano treatment.

Another important key element in the apoptotic process is the P53 gene, whose protein promotes BAX gene expression by direct activation of its promoter and BCL2 downregulation [111,112,113]. P53 is activated in response to a wide range of genotoxic insults. It is involved in several DNA-repair machineries, such as nucleotide excision repair, for the removal of helix-distorting lesions (typical of UV-damage) and base excision repair (BER) in case of base oxidative modifications [114,115]. As known in the literature, plant compounds, especially polyphenols, can cause oxidative DNA strand breakage, alone or in the presence of transition metal ions. Indeed, among all, the copper (that is the most abundant ion of the cell nucleus, together with zinc), after reduction from Cu(II) to Cu(I) form by the action of plant metabolites, is particularly prone to produce ROS (especially the hydroxyl radical) during its re-oxidation, bind chromatin (particularly guanines), and cause DNA breakages [116,117].

The existence of such types of event prompted us to analyze P53 mRNA and protein levels and evaluate DNA damages (detecting γH2AX and 53BP1 foci by IF) upon HCOE10 exposure. Oregano treatment induced in A375 p53 upregulation, at both the transcriptional and translational level, and DNA breakages. In detail, γH2AX is the phosphorylated form of the histone 2AX (H2AX). Its phosphorylation is an early consequence of double and single-strand breakages [118,119,120,121]; therefore, the detection of γH2AX is widely used as a marker of DNA damage. On the other hand, p53 binding protein 1 (53BP1) locates only to DNA double-strand breaks [122]. IF analyses highlighted a great number of γH2AX and 53BP1 foci, which often co-localized in HCOE10-treated cells with respect to untreated controls. This evidence, together with Ames test results, pointed out that oregano hydroalcoholic extract acts as a genotoxic but not mutagenic agent in A375 melanoma cells, causing DNA single and double-strand breaks [123]. Furthermore, taking into account that DNA damage often results from the binding of phytochemical to transition metal ions, such as copper [124,125,126], co-treatments with HCOE10 and a copper chelator (TETA) were performed. Surprisingly, the sequestration of copper by TETA protected A375 cells from HCOE10-induced DNA damage, confirming that this metal ion plays a fundamental role in oregano bioactivity and relative DNA cleavage reaction.

In cancer masses, the concentration of iron and zinc is lower, whereas the copper concentration is usually higher than in healthy tissues [127,128,129,130,131,132]. This feature can explain why plant compounds exert selective cytotoxic activity against tumor cells but not toward non-tumor ones [133,134,135,136,137,138,139], justifying our data about oregano cytotoxicity on B16–F10 and A375 but not on C2C12.

In conclusion, *O. vulgare* hydroalcoholic extract, due to its peculiar chemical profile and pro-oxidant effect, inhibits melanogenesis and melanoma cell proliferation. Through in-depth molecular analyses, the antineoplastic activity of the oregano extract was associated to its ability to trigger programmed cell death (apoptosis and necroptosis) in A375 human melanoma cells via mitochondria and DNA damage. As this molecular mechanism was correlated to the intracellular/nuclear concentration of copper ions, oregano phytochemicals appeared to be slightly toxic or non-toxic for non-tumor cells. All this evidence represents a robust starting point for further studied focused on the design of new anti-melanoma natural drugs. Indeed, according to the present data, *O. vulgare* phytocomplex, working in synergy, represent an excellent candidate as anticancer agent, being highly selective and effective against human melanoma cells.

## Figures and Tables

**Table 1:** High-performance liquid chromatography-diode array detector (HPLC-DAD) profiles of oregano extract. Plant molecules and their concentration, detected in hydroalcoholic oregano extract (HCOE) by HPLC-DAD analysis, are reported. Results were indicated as µg of metabolite per 100 mg of dried material (µg/100 mg DMW) and represent the mean ± SD of six independent experiments.

Compound	µg/100 mg DMW ± SD
Chrysin	8.47 ± 0.06
Rutin	2.15 ± 0.05
Myricetin	0.03 ± 0.01
Caffeic acid	0.35 ± 0.02
1,1-Dimethylallyl caffeate	1.28 ± 0.03
Caffeic acid phenethyl ester	0.76 ± 0.03
Gallic acid	0.18 ± 0.01
Kaempferol	0.04 ± 0.01
*p*-Coumaric acid	0.30 ± 0.01
Genistein	1.02 ± 0.02
Quercetin-3-*o*-arabinoside	2.37 ± 0.04
Chlorogenic acid	1.03 ± 0.04
Apigenin	0.26 ± 0.01

**Table 2:** GC-MS profile of oregano extract. Plant metabolites and their relative abundance, detected in HCOE by GC-MS analysis, are reported. The relative abundance of each molecule was indicated as a percentage value with respect to the total mixture (100%). Values represented the mean of three independent experiments. The SD for each measurement was always <5% of the respective molecule peak area.

GC-MS Detected Compound	%
*m*-Cymol	0.57
*p*-Mentha-1,3,8-triene	0.31
*p*-Cymene-2,5-dione	0.87
Thymol	16.64
Carvacrol	34.82
Caryophyllene oxide	0.90
*t*-Butylhydroquinone	1.86
Isopropyl laurate	0.94
Palmitic acid	2.50
Ethyl palmitate	1.33
Phytol	0.84
Retinoic acid	0.73
Methyl linolenate	7.96
Ethyl linolenate	7.35
3,3-Dimethylbutanoic acid	0.03
*p*-Mentha-1,4-diene	0.13
*alpha*-Aminoisobutanoic acid	0.03
*p*-Mentha-6,8-dien-2-ol	0.94
Myrtenyl acetate	0.54
2,6-Dimethyl-1,3,5,7-octatetraene	0.29
3,5-Dihydroxy-6-methyl-2,3-dihydro-4H-pyran-4-one	0.33
Thujone	0.52
Octyl acetate	0.19
2,3-Dimethyl-2-pentanol	0.11
Dimethyl malonate	0.04
Ehtyl acetimidate	0.09
Dimethylhexynediol	0.20
Linalool oxide	0.89
*trans*-2-Hexenyl caproate	0.29
2-Ethyl-3-hydroxyhexyl 2-methylpropanoate	0.09
1-Tetradecanol	0.10
Hydroxydehydrostevic acid	0.11
2,2,4-Trimethyl-1,3-pentanediol diisobutyrate	1.22
Caprylic ether	0.07
*alpha*-Methylglucoside	7.95
Hexadecane	0.92
Retinyl acetate	0.84
Stearic acid	0.99
Butyl citrate	3.58
Jasmone	0.44
Oleic Acid	0.33
Oleic acid amide	0.72
3-Hexadecanol	0.40

**Table 3:** Cytotoxicity analysis. The percentages of C2C12, B16-F10, and A375 dead cells identified by the Trypan Blue test after 24 and 48 h of HCOE treatment are reported. Data are reported as mean ± SD of four independent replicates. (* *p* < 0.05 vs. control).

Cell Line	Treatment		Time	
			24 h	48 h
**C2C12**	**CNT (PBS)**		6.07 ± 0.80	4.61 ± 0.11
	**HCOE**	(2 mg/mL)	13.33 ± 0.41	11.67 ± 2.60
		(4 mg/mL)	13.16 ± 0.32	11.33 ± 3.43
		(6 mg/mL)	18.86 ± 0.28	16.35 ± 0.80
		(8 mg/mL)	20.25 ± 0.51 *	19.78 ± 1.57 *
		(10 mg/mL)	20.67 ± 0.17 *	21.25 ± 2.36 *
**B16-F10**	**CNT (PBS)**		1.86 ± 0.13	5.31 ± 0.26
	**HCOE**	(2 mg/mL)	14.90 ± 1.16	14.84 ± 1.17
		(4 mg/mL)	12.98 ± 2.38	26.62 ± 0.95 *
		(6 mg/mL)	24.36 ± 0.84 *	33.29 ± 1.49 *
		(8 mg/mL)	37.30 ± 2.90 *	39.45 ± 3.44 *
		(10 mg/mL)	42.00 ± 3.73 *	44.75 ± 1.86 *
**A375**	**CNT (PBS)**		5.38 ± 1.09	5.00 ± 1.71
	**HCOE**	(2 mg/mL)	17.78 ± 1.38	13.10 ± 1.92
		(4 mg/mL)	16.82 ± 0.75	25.38 ± 2.96 *
		(6 mg/mL)	29.44 ± 1.17 *	34.71 ± 1.86 *
		(8 mg/mL)	31.21 ± 3.81 *	48.26 ± 0.77 *
		(10 mg/mL)	47.14 ± 1.73 *	55.13 ± 1.47 *

## Supplementary Materials

The following are available online at https://www.mdpi.com/2304-8158/9/10/1486/s1, Figure S1: HPLC analysis; Table S1: RT-qPCR primers; Table S2: Ames test for mutagenic analysis; Table S3: Ames test for mutagen-protective analysis.
