# Supplementary material for: Oregano Phytocomplex Induces Programmed Cell Death in Melanoma Lines via Mitochondria and DNA Damage
Source: Foods. 2020 Oct 17;9(10):1486. doi: 10.3390/foods9101486 (PMC7603152; doi:10.3390/foods9101486)
Supplement: Supplementary file 1 [file foods-09-01486-s001.pdf]

TABLE S1 – Nanni et al.

Gene name	Primers	References
MITF	F: 5'-CTGGAAATGCTAGAATACAG-3'<br>R: 5'-TCTTCTTCTTCGTTCAATCA-3'	Mansky et al., 2002
TYR	F: 5'-ATTGATTTTGCCCATGAAGC-3'<br>R: 5'-CCCAGATCCTTGGATGTTATG-3'	Vetrini et al., 2004
TYRP1	F: 5'-ACTGACCCTTGTGGCTCATC-3'<br>R: 5'-GAGAAATCCACATCCCCAAA-3'	Vetrini et al., 2004
P21	F: 5'- AGACCAGCATGACAGATTTC-3'<br>R: 5'- ACTGAGACTAAGGCAGAAGA-3'	Zhang et al., 2017
P27	F: 5'- ATAAGGAAGCGACCTGCAAC-3'<br>R: 5'- ACGTTTGACGTCTTCTGAGG-3'	Zhang et al., 2017
P53	F: 5'- GTGAGCGCTTCGAGATGTTC-3'<br>R: 5'- CCCTTCTGTCTTGAACATGAG-3'	Zhang et al., 2017
CCNB1	F: 5'- GACCTGTGTCAGGCTTTCTCTG-3'<br>R: 5'- GGTATTTTGGTCTGACTGCTTGC-3'	Lin et al., 2017
CDK1	F: 5'- GGAAACCAGGAAGCCTAGCATC-3'<br>R: 5'- GGATGATTCAGTGCCATTTTGCC-3'	Lin et al., 2017
ACTB	F: 5'-ACCACCATGTACCCTGGCATT-3'<br>R: 5'-CCACACGGAGTACTTGCGCTCA-3'	Simard and Chabot, 2000

Table S1. RT-qPCR primers. The primer sequences used in RT-qPCR analysis and relative references were reported.

References

Mansky, K.C., Sulzbacher, S., Purdom, G., Nelsen, L., Hume, D.A., Rehli, M., & Ostrowski, M.C. (2002). The microphthalmia transcription factor and the related helix-loop-helix zipper factors TFE-3 and TFE-C collaborate to activate the tartrate-resistant acid phosphatase promoter. *J Leukoc Biol*, *71*, 304-310.

Vetrini, F., Auricchio, A., Du, J., Angeletti, B., Fisher, D.E., Ballabio, A., & Marigo, V. (2004). The microphthalmia transcription factor (Mitf) controls expression of the ocular albinism type 1 gene: link between melanin synthesis and melanosome biogenesis. *Mol Cell Biol*, *24*, 6550-6559.

Zhang, Y., Yuan, Y., Liang, P., Guo, X., Ying, Y., Shu, X.S., Gao, M.Jr., & Cheng, Y. (2017). OSR1 is a novel epigenetic silenced tumor suppressor regulating invasion and proliferation in renal cell carcinoma. *Oncotarget*, *8*, 30008-30018.

Lin, Y.T., Lin, C.C., Wang, H.C., & Hsu, Y.C. (2017). Induction of mitotic delay in pharyngeal and nasopharyngeal carcinoma cells using an aqueous extract of *Ajuga bracteosa*. *Int J Med Sci*, *14*, 462-469.

Simard, M.J., & Chabot, B. (2000). Control of hnRNP A1 alternative splicing: an intron element represses use of the common 3' splice site. *Mol Cell Biol*, *20*, 7353-7362.

TABLE S2 – Nanni et al.

Strain	Concentration (mg/plate)	- S9		+S9	
		No. Colonies ± SD	t/c	No. Colonies ± SD	t/c
TA97A	DMSO	112.3 ± 8.08	1.0	135.0 ± 8.19	1.0
	C+	424.3 ± 21.73	3.8	607.7 ± 25.70	4.5
	0.1	150 ± 1.41	1.3	150 ± 7.07	1.1
	0.07	157 ± 4.24	1.4	132.5 ± 6.36	1.0
	0.05	129.5 ± 2.12	1.2	124.5 ± 13.44	0.9
	0.03	130 ± 4.24	1.2	145.5 ± 6.36	1.1
	0.01	124.5 ± 10.61	1.1	137 ± 7.07	1.0
TA98	DMSO	25.0 ± 4.58	1.0	35.3 ± 6.66	1.0
	C+	92.3 ± 8.50	3.7	718.7 ± 64.70	20.3
	0.1	35.5 ± 3.54	1.4	45.5 ± 2.12	1.3
	0.07	35 ± 1.41	1.4	43.5 ± 3.54	1.2
	0.05	29.5 ± 2.12	1.2	37.5 ± 3.54	1.1
	0.03	26.5 ± 0.71	1.1	36.5 ± 0.71	1.0
	0.01	25.5 ± 0.71	1.0	37.5 ± 0.71	1.1
TA100	DMSO	95.3 ± 7.37	1.0	116.3 ± 8.74	1.0
	C+	617.7 ± 15.50	6.5	525.7 ± 25.58	4.5
	0.1	97.5 ± 2.12	1.0	157 ± 1.41	1.3
	0.07	108.5 ± 4.95	1.1	140 ± 8.49	1.2
	0.05	117 ± 8.49	1.2	132 ± 5.66	1.1
	0.03	94 ± 5.66	1.0	146 ± 15.56	1.3
	0.01	89.5 ± 2.12	0.9	127 ± 1.41	1.1
TA1535	DMSO	20.3 ± 3.06	1.0	14.3 ± 2.08	1.0
	C+	122.0 ± 5.57	6.0	92.7 ± 7.37	6.5
	0.1	26.5 ± 2.12	1.3	16.5 ± 0.71	1.2
	0.07	24.5 ± 0.71	1.2	15.5 ± 0.71	1.1
	0.05	23.5 ± 0.71	1.2	17 ± 1.41	1.2
	0.03	20 ± 2.83	1.0	14.5 ± 0.71	1.0
	0.01	20 ± 1.41	1.0	14 ± 1.41	1.0

Table S2. Ames test for mutagenic analysis. TA97a, TA98, TA1535 and TA100 were treated with different concentration of HCOE, with and without metabolic activator (+S9 mix, -S9 mix, respectively). Negative control was represented by DMSO treatment. Positive controls (C+), in absence of S9 mix, were represented by 2-nitrofluorene for TA97a, TA98 and TA1535 strains and by sodium azide for TA100 strains, while, in presence of S9 mix, by 2-aminoanthracene for all *Salmonella* strains. Results are expressed as average number of colonies per plate ± SD T/c is the ratio between the number of *Salmonella* colonies grown in presence of HCOE and those of the negative controls (C-).

TABLE S3 – Nanni et al.

Strain	Concentration (mg/plate)	- S9		+S9	
		No. Colonies ± SD	IR (%)	No. Colonies ± SD	IR (%)
TA97A	DMSO	137.7 ± 5.51	--	145.7 ± 9.07	--
	C	540.0 ± 13.45	--	638.0 ± 18.03	--
	0.1	542.5 ± 17.68	-0.6	628.5 ± 6.36	1.9
	0.07	534.5 ± 9.19	1.4	644 ± 18.38	-1.2
	0.05	545 ± 2.83	-1.2	642 ± 4.24	-0.8
	0.03	521 ± 4.24	4.7	675 ± 35.36	-7.5
	0.01	517 ± 8.49	5.7	660.5 ± 7.78	-4.6
TA98	DMSO	29.3 ± 1.53	--	38.0 ± 2.00	--
	C	139.7 ± 6.81	--	612.0 ± 14.00	--
	0.1	144.5 ± 2.12	-4.4	618.5 ± 4.95	-1.1
	0.07	141 ± 4.24	-1.2	656.5 ± 13.44	-7.8
	0.05	135.5 ± 7.78	3.8	605.5 ± 7.78	1.1
	0.03	144 ± 8.49	-3.9	596 ± 9.90	2.8
	0.01	133.5 ± 4.95	5.6	597 ± 2.83	2.6
TA100	DMSO	131.7 ± 4.04	--	121.7 ± 3.79	--
	C	581.7 ± 4.04	--	486.0 ± 12.12	--
	0.1	625.5 ± 7.78	-9.7	483 ± 9.90	0.8
	0.07	609.5 ± 38.89	-6.2	476.5 ± 4.95	2.6
	0.05	577.5 ± 16.26	0.9	499 ± 46.67	-3.6
	0.03	607.5 ± 50.20	-5.7	460 ± 4.24	7.1
	0.01	589 ± 1.41	-1.6	474.5 ± 37.48	3.2
TA1535	DMSO	19.7 ± 1.15	--	11.7 ± 1.53	--
	C	144.0 ± 7.00	--	105.0 ± 7.55	--
	0.1	138.5 ± 3.54	4.4	115.5 ± 6.36	-11.3
	0.07	148.5 ± 6.36	-3.6	120 ± 2.83	-16.1
	0.05	142.5 ± 3.54	1.2	105.5 ± 2.12	-0.5
	0.03	149 ± 1.41	-4.0	102 ± 1.41	3.2
	0.01	134 ± 1.41	8.0	103.5 ± 3.54	1.6

Table S3. Ames test for mutagen-protective analysis. TA97a, TA98, TA1535 and TA100 were treated with mutagen agents, with and without metabolic activator (+S9 mix, -S9 mix, respectively), in absence (C) or presence of different HCOE concentrations. Negative control was represented by DMSO treatment. In absence of S9 mix, TA97a, TA98 and TA1535 strains were treated by 2-nitrofluorene, whereas TA100 strain was incubated with sodium azide. In presence of S9 mix, all *Salmonella* strains were exposed to 2-aminoanthracene. Results are expressed as average of number of colonies per plate ± SD IR (%) represents the percentage of inhibition ratio. Positive values indicate protective activity.

FIGURE S1 – Nanni et al.

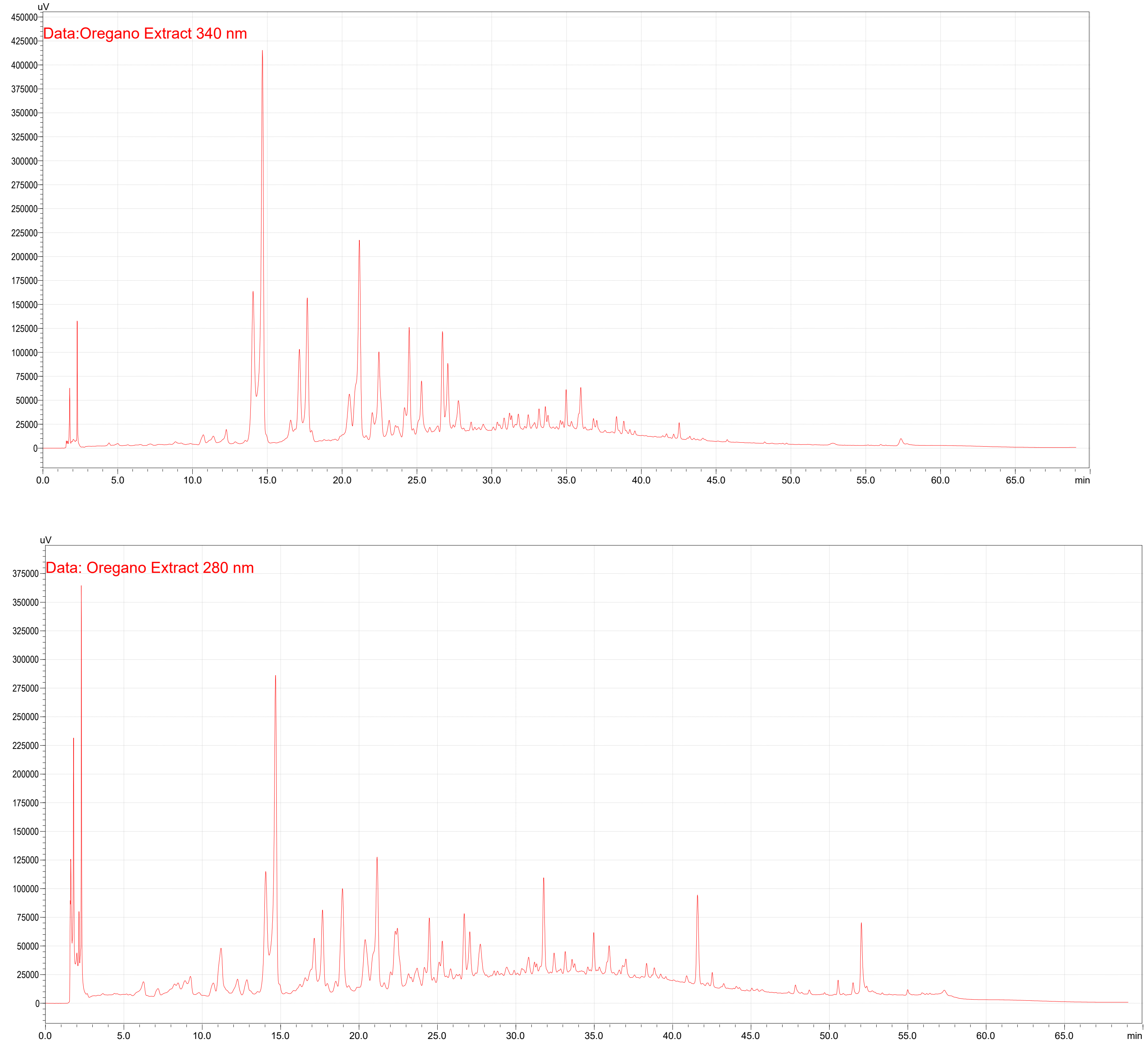

FIGURE S1. HPLC-DAD qualitative analysis. HPLC-DAD profiles of oregano extract acquired at 340 nm (absorption wavelength of flavonoids; upper panel) and 280 nm (absorption wavelength of phenols; lower panel).
